# Draft genome sequence of *Actinotignum schaalii* DSM 15541^T^: Genetic insights into the lifestyle, cell fitness and virulence

**DOI:** 10.1371/journal.pone.0188914

**Published:** 2017-12-07

**Authors:** Atteyet F. Yassin, Stefan Langenberg, Marcel Huntemann, Alicia Clum, Manoj Pillay, Krishnaveni Palaniappan, Neha Varghese, Natalia Mikhailova, Supratim Mukherjee, T. B. K. Reddy, Chris Daum, Nicole Shapiro, Natalia Ivanova, Tanja Woyke, Nikos C. Kyrpides

**Affiliations:** 1 Institut für medizinische Mikrobiologie und Immunologie der Universität Bonn, Bonn, Germany; 2 Klinik und Poliklinik für Hals-Nasen-Ohrenheilkunde/Chirurgie, Bonn, Germany; 3 Department of Energy Joint Genome Institute, Genome Biology Program, Walnut Creek, CA, United States of America; Universite Paris-Sud, FRANCE

## Abstract

The permanent draft genome sequence of A*ctinotignum schaalii* DSM 15541^T^ is presented. The annotated genome includes 2,130,987 bp, with 1777 protein-coding and 58 rRNA-coding genes. Genome sequence analysis revealed absence of genes encoding for: components of the PTS systems, enzymes of the TCA cycle, glyoxylate shunt and gluconeogensis. Genomic data revealed that *A*. *schaalii* is able to oxidize carbohydrates via glycolysis, the nonoxidative pentose phosphate and the Entner-Doudoroff pathways. Besides, the genome harbors genes encoding for enzymes involved in the conversion of pyruvate to lactate, acetate and ethanol, which are found to be the end products of carbohydrate fermentation. The genome contained the gene encoding Type I fatty acid synthase required for *de novo* FAS biosynthesis. The *plsY* and *plsX* genes encoding the acyltransferases necessary for phosphatidic acid biosynthesis were absent from the genome. The genome harbors genes encoding enzymes responsible for isoprene biosynthesis via the mevalonate (MVA) pathway. Genes encoding enzymes that confer resistance to reactive oxygen species (ROS) were identified. In addition, *A*. *schaalii* harbors genes that protect the genome against viral infections. These include restriction-modification (RM) systems, type II toxin-antitoxin (TA), CRISPR-Cas and abortive infection system. *A*. *schaalii* genome also encodes several virulence factors that contribute to adhesion and internalization of this pathogen such as the *tad* genes encoding proteins required for pili assembly, the *nanI* gene encoding exo-alpha-sialidase, genes encoding heat shock proteins and genes encoding type VII secretion system. These features are consistent with anaerobic and pathogenic lifestyles. Finally, resistance to ciprofloxacin occurs by mutation in chromosomal genes that encode the subunits of DNA-gyrase (GyrA) and topisomerase IV (ParC) enzymes, while resistant to metronidazole was due to the *frxA* gene, which encodes NADPH-flavin oxidoreductase.

## Introduction

The genus *Actinotignum* was introduced by [1] to accommodate bacterial strains which are associated with human infections and previously classified in the genus *Actinobaculum*. The genus belongs to the family *Actinomycetaceae* of the class *Actinobacteria*. Currently, the genus comprises three validly published species: *A*. *sanguinis*, *A*. *schaalii* and *A*. *urinale*.

*A*. *schaalii* resides as a commensal in the human genital and urinary tract [2]. It is an opportunistic pathogen associated with urinary tract infections (UTI), bloodstream infections, endocarditis, abscess formation, Fournier’s gangrene and vertebral osteomyelitis, predominantly in elderly patients [3, 4, 5, 6].

*A*. *schaalii* has the typical morphological, physiological and chemotaxonomic characteristics observed in other members of the genus *Actinotignum*. The cells are Gram-positive, non-motile, nonspore-forming and straight to slightly curved rods. The organism is anaerobic or facultatively anaerobic, grows well under anaerobic conditions, poorly in an atmosphere of 5% CO_2_ and failed to grow under aerobic conditions, catalase- and oxidase-negative. Metabolism is fermentative. In Peptone-Yeast extract-Glucose (PYG) broth, glucose is fermented to L-lactic acid, acetic acid and ethanol. The organism is able to hydrolyze hippurtae. Nitrate is not reduced to nitrite. *A*. *schaalii* ferments glucose, L-arabinose, maltose, D-ribose, sucrose and xylose. The organism is positive for α-glucosidase and leucine arylamidase activities and negative for the other enzyme activities tested by the API ZYM (bioMérieux) panel. *A*. *schaalii* is susceptible to ampicillin (MIC 0.016 μg/ml), clindamycin (MIC 0.016 μg/ml), gentamicin (MIC 0.75 μg/ml), vancomycin (MIC 0.19 μg/ml), linezoliod (MIC 0.125 μg/ml), co-trimoxazole (MIC <0.002 μg/ml) and rifampicin (MIC <0.002 μg/ml), but resistance to metronidazole (MIC >256 μg/ml) and ciprofloxacin (MIC ≥2 μg/ml). The polar lipids include diphosphatidylglycerol, phosphatidylglycerol, phosphatidylinositol and a choline-containing phospholipid (AbGl). The major cellular fatty acids are C_16:0_, C_18:0_, and C_18:1_ω9c. The diagnostic whole-cell sugars include glucose, rhamnose and 6-deoxytalose. The peptidoglycan is of type A5α, variation L-Lys-L-Lys-D-Glu. The DNA G+C content initially reported with 57 mol% [7], was much lower than the 62.2% inferred from the genome sequence.

Advances in sequencing technology now facilitate the rapid determination of whole genome sequencing (WGS) in microbial species. WGS enables: the study of metabolic pathways [8], development of novel diagnostic tests [9], detection of drug targets and antibiotic resistance genes [10], identification of potential virulence determinants [11] and development of new vaccines [12]. In this study, we report on the draft genome sequence of *Actinotignum schaalii* DSM 15541^T^ to gain insights into its metabolic pathways and to assess the link between the observable phenotypes and the genes and the metabolic pathways. A comparative genomic analysis among available genomes of *A*. *schaalii* strains e.g., genomes of *A*. *schaalii* CCUG 27420^T^ deposited under the accession number CP008802 [13] and *A*. *schaalii* FB123-CAN-2 (AGWM00000000), was performed to help identification of orthologous genes. The strain was selected for genome sequencing on the basis of its phylogenetic position, and is part of the Genomic Encyclopedia of Type Strains, Phase I (K. MG-I) [14, 15]. KMG-I is the first of the production phases of the GEBA: sequencing a myriad of type strains initiative [16] and a Genomic Standards Consortium project [17].

## Materials and methods

### Genome sequencing and assembly

Genomic DNA was prepared by using the MasterPure^TM^ Gram Positive DNA Purification Kit (Epicentre MGP04100) after modification of the standard protocol provided by the manufacturer as described previously [18]. The draft genome of *Actinobaculum schaalii* DSM 15541^T^ was generated at the DOE Joint genome Institute (JGI) using the Illumina technology [19]. An Illumina std shotgun library was constructed and sequenced using the Illumina HiSeq 2000 platform which generated 8,122,984 reads totaling 1,218.4 Mb. All general aspects of library construction and sequencing performed at the JGI can be found at http://www.jgi.doe.gov. All raw Illumina sequence data was passed through DUK, a filtering program developed at JGI, which removes known Illumina sequencing and library preparation artifacts [20]. Following steps were then performed for assembly: (1) filtered Illumina reads were assembled using Velvet (version 1.1.04) [21], (2) 1–3 kb simulated paired end reads were created from Velvet contigs using wgsim [22], (3) Illumina reads were assembled with simulated read pairs using Allpaths–LG (version r42328) [23]. Parameters for assembly steps were: 1) Velvet (velveth: 63 –shortPaired and velvetg:–very clean yes–exportFiltered yes–min contig lgth 500 –scaffolding no–cov cutoff 10) 2) wgsim (–e 0–1 100–2 100 –r 0 –R 0 –X 0) 3) Allpaths–LG (PrepareAllpathsInputs: PHRED 64 = 1 PLOIDY = 1 FRAG COVERAGE = 125 JUMP COVERAGE = 25 LONG JUMP COV = 50, RunAllpathsLG: THREADS = 8 RUN = std shredpairs TARGETS = standard VAPI WARN ONLY = True OVERWRITE = True). The final draft assembly contained 29 contigs in 27 scaffolds. The total size of the genome is 2.1 Mb and the final assembly is based on 259.0 Mb of Illumina data, which provides an average 121.6X coverage of the genome.

### Genome annotation

Genes were identified using Prodigal [24], followed by a round of manual curation using GenePRIMP [25] for finished genomes and Draft genomes in fewer than 10 scaffolds. The predicted CDSs were translated and used to search the National Center for Biotechnology Information (NCBI) nonredundant database, UniProt, TIGRFam, Pfam, KEGG, COG, and InterPro databases. The tRNAScanSE tool [26] was used to find tRNA genes, whereas ribosomal RNA genes were found by searches against models of the ribosomal RNA genes built from SILVA [27]. Other non–coding RNAs such as the RNA components of the protein secretion complex and the RNase P were identified by searching the genome for the corresponding Rfam profiles using INFERNAL [28]. Additional gene prediction analysis and manual functional annotation was performed within the Integrated Microbial Genomes (IMG) platform [29] developed by the Joint Genome Institute, Walnut Creek, CA, USA [30].

### Phylogenetic analyses

Phylogenetic analyses of 16S rRNA gene sequences were performed using the ARB-package [31]. Evolutionary distances were calculated using the method [32]. Phylogenetic trees were reconstructed using the neighbour-joining [33], maximum-likelihood (RAxML; [34]) and maximum-parsimony (ARB_PARS) methods as implemented in the ARB package. The topology of the neighbour-joining tree was evaluated using bootstrap analyses [35] based on 1000 resamplings. The sequence of the single 16S rRNA gene copy (1349 nucleotides) in the genome of *A*. *schaalii* DSM 15541^T^ was added to the ARB database [31] and compared with the 16S rRNA gene sequences of the type strains of *Actinotignum* species obtained from the NCBI database. This sequence is identical to the previously published 16S rRNA sequence (AM922112).

### Nucleotide sequence accession numbers

The draft genome sequence of *A*. *schaalii* DSM 15541^T^ has been deposited at the DDBJ/EMBL/GenBank under the accession number AUBK00000000. The version described in this paper is version AUBK00000000.1.

## Results and discussion

### General genome features

**Fig 1:**
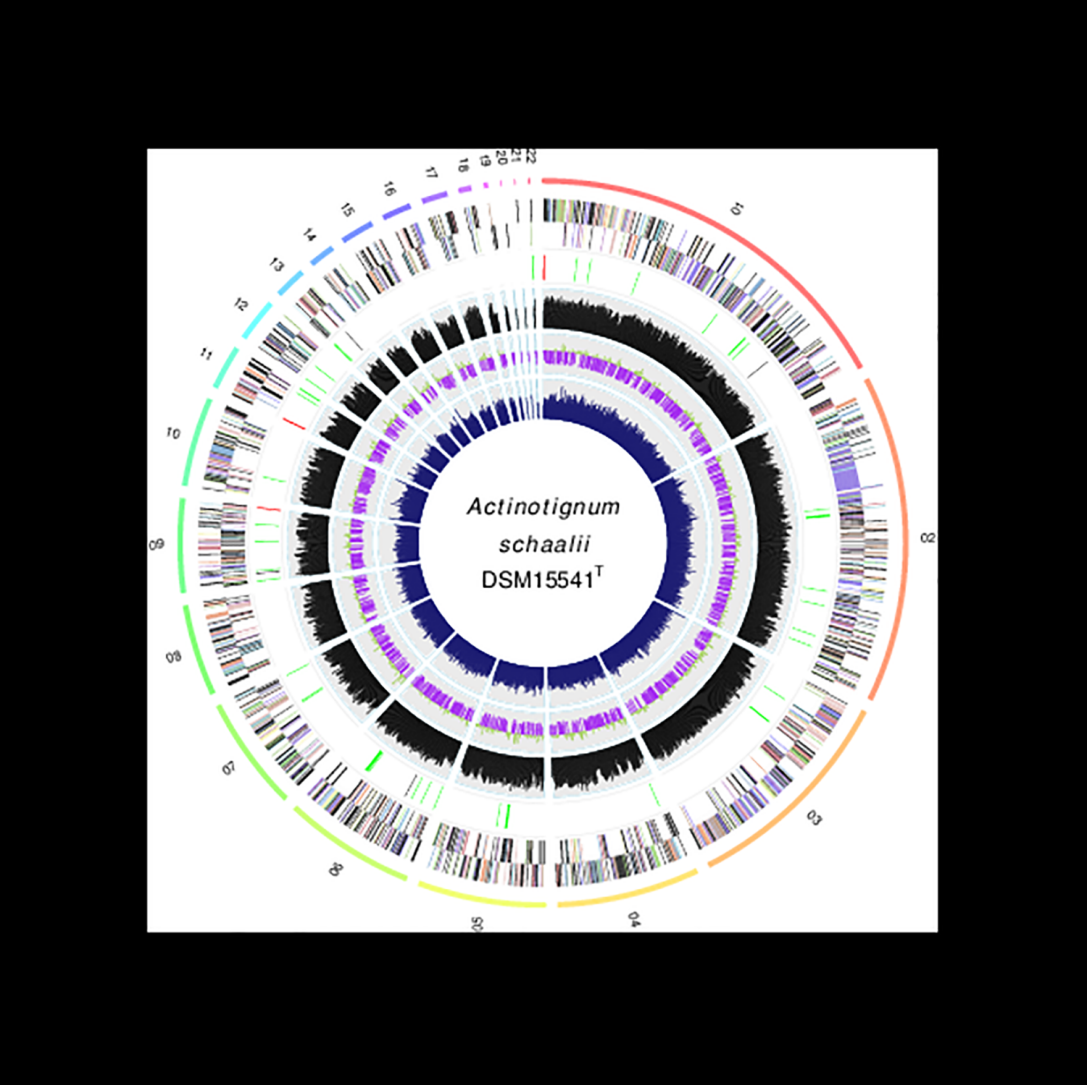
Circular representation of *A*. *Schaalii* DSM15541^T^ genome (27 scaffolds). Features from the outer circle to the center are: genes on the forward strand (color by COG categories), genes on the reverse strand (color by COG cataegories), RNA genes (tRNA green, rRNA red, other RNAs black), % G+C content, GC skew (purple/olive) and codon adaptation index. The figure was obtained using OmicCircos [36].

The genome sequences of *A*. *schaalii* strains DSM 15541^T^ (= CCUG27420^T^) and FB 123-CAN-2 are relatively similar in size. The genome of *A*. *schaalii* DSM 15541^T^ consists of 2,130,987 bp in length with an average G+C content of 62.25 mol% (Fig 1).

The origin of replication (*oriC*) was identified in a region (710 bp) located between the *dnaA* and the *dnaN* genes. The predicted *oriC* is flanked on one side by the *dnaA* (G444DRAFT_01293), *dnaN* (G444DRAFT_01294), *recF* (G444DRAFT_01295), *gyrB* (G444DRAFT_01298) and *gyrA* (G444DRAFT_01299) genes and on the other side by *parB* (G444DRAFT_01288), *parA* (G444DRAFT_01289), *gidB* (G444DRAFT_01290), *spoIIIJ* (G444DRAFT_01291) and *yidC* (G444DRAFT_01292) genes. The genome contains 1835 predicted genes, of which 1777 (96.84%) were protein-coding genes and 58 (3.16%) were rRNA coding genes. Among the protein-coding genes, 1371 genes (74.71%) were assigned putative biological functions and the rest 406 genes (22.13%) were hypothetical proteins of unkown function. Most of the protein-coding genes, 1177 (64.14%), were assigned to Clusters of Orthologous Groups (COG). Of the 58 RNA genes, eight are rRNA genes, three are 5S rRNA genes, four are 23S rRNA genes, one is 16S rRNA genes, fourty-six are tRNA genes and four other RNA genes. The genome contains two clustered regularly interspaced short palindromic repeat (CRISPR) loci. A summary of the genome properties and statistics are given in Table 1.

**Table 1:** General genome characteristics of *Actinotignum schaalii* DSM 15541^T^.

Feature	Value	% of Total
Genome size (bp)	2,130,987	100
DNA coding region (bp)	1,889,798	88.68
DNA G+C content (bp)	1,326,485	62.25
DNA scaffolds	27	100
Total genes	1, 835	100
RNA genes	58	3.16
Protein-coding genes	1,777	96.84
Genes with function prediction (proteins)	1,371	74.71
Genes in paralog clusters	457	21.18
Genes assigned to COGs	1,177	64.14
Genes with Pfam domains	1,411	76.89
Genes with signal peptides	127	6.92
Genes with transmembrane helices	490	26. 70
CRISPR repeats	2	

### Taxonomy and phylogeny

**Fig 2:**
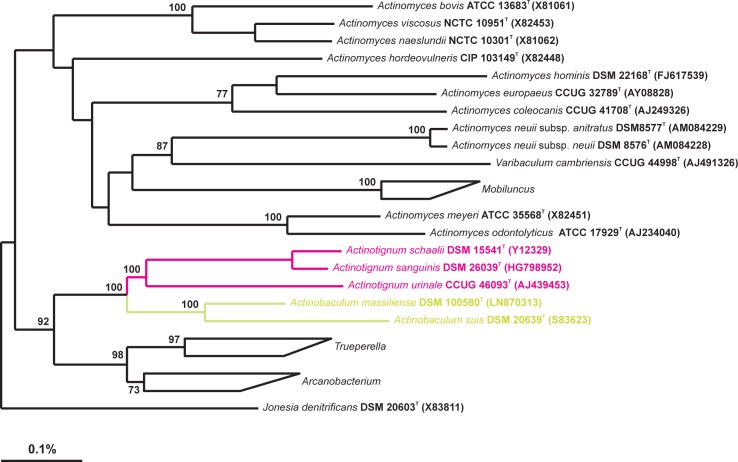
Neighbour-joining phylogenetic tree based on 16S rRNA gene sequences. The tree showing the phylogenetic position of *A*. *schaalii* DSM 15441^T^, the type strains of other species of the genus *Actinotignum* and other related members of the genera of the family *Actinomycetaceae*. *Jonesia denitrificans* was used as outgroup. Bootstrap values (>70%), expressed as a percentage of 1000 replication, are indicated at the nodes. Bar, 0.1% substitution per nucleotide position.

A tree constructed using the neighbour-joining method depicted the pylogenetic position of *A*. *schaalii* is shown in Fig 2. The topology of the resultant tree was similar to the topology of the trees constructed by the maximum-likelihood and maximum parsimony algorithms. All the dendrograms were similar in that *Actinotignum* species formed a well separated clade (100% bootstrap value) within the radiation of the family *Actinomycetaceae*. In each of the dendrograms calculated *A*. *schaalii* and *A*. *sanguinis* are phylogenetic neighbours. On the basis of 16S rRNA gene sequence similarity *A*. *schaalii* was most closely related to *A*. *sanguinis* (98.5% sequence similarity). The phylogenetic relatedness and the high degree of 16S rRNA gene sequence similarity between *A*. *schaalii* and *A*. *sanguinis* is not unexpected as they are ecologically similar. They reside as commensal microbiota of the human urinary tract and have the potential to cause a wide spectrum of diseases in human such as urinary tract infections and bacteremia.

It should be noted that in all trees *Actinotignum* appeared as sister clade of the *Actinobaculum* lineage, a situation already depicted in the original description of the genus *Actinotignum* [1]. Apart from the lower 16S rRNA gene sequence similarities (less than 93.2%) and differences in the chemotaxonomic properties, members of the two genera are ecologically distinct. *Actinobaculum suis* is a resident of the urinary tract of sows and has never been isolated from human speciemens. Although *A*. *massiliense* and *A*. *suis* share the same clade in the phylogenetic tree, the former species is restricted to the human urinary tract and still await taxonomic revision. However, both species are distinguishable (at this time) on the basis of the G+C content (*A*. *massiliense* is 60.1 mol% compared with 57.8 mol% for *A*. *suis*) and habitat.

### Carbohydrate transport and metabolism

#### Sugar transport systems

A bioinformatic reconstruction of the central carbon metabolism of *A*. *schaalii* DSM 15541^T^ revealed the absence of genes coding for components of the phosphoenolpyruvate:phosphotransferase systems (PTSs). A single homologue of a β-glucosidic-specific IIC permease (G444DRAFT_01249) was found. This IIC permease cannot function via a PTS-dependent mechanism since the organism lacks all other PTS protein homologues, including EI and HPr. A similar case was reported in *Bacteroides thetaiotaomicron*, which encodes a single homologue of a galacticol IIC protein and lacks PTS proteins completely [37]. *A*. *schaalii* also lacks a complete dihydroxyacetone PTS (DHA PTS). The genome harbors the *dhaK* and *dhaL* genes encoding DhaK (G444DRAFT_00672) and DhaL (G444DRAFT_00673) proteins, respectively, but lacks the *dhaM* gene encoding for DhaM homologue. Like *Methylococcus capsulata*, which has DhaK and DhaL but no other PTS protein [37], it seems likely that *A*. *schaalii* cannot phosphorylate DHA with either PEP or ATP.

On the other hand, genome sequence analysis revealed the presence of genes predicted to encode primary sugar transporters of the ATP-binding cassette (ABC) and secondary transporters of the major facilitator (MFS) superfamilies. A characteristic feature of the ABC transporter is that the genes for three components: the ATP-binding protein, the membrane protein and the substrate-binding protein, frequently form an operon [38].Two families within the ABC superfamily concerned exclusively with carbohydrate uptake are present in the genome of *A*. *schaalii*. The carbohydrate uptake transporter-1 (CUT1) and -2 (CUT2) families exhibit specificity for mono-/di-/oligosaccharides and monosaccharides, respectively [39]. Three CUT1 (TC3.A.1.1.-) transporters were found in *A*. *schaalii* genome including homologues of the structural genes *malEFGK* encoding four essential constituents of the maltose transporter subunits MalEFGK involved in maltose transport (S1 Fig), homologues of the structural genes *msmEFGK* encodiong subunits of the multiple-sugar metabolism transporter MsmEFGK involved in the uptake and metabolism of disaccharides and/or oligosaccharides (S2 Fig). Although the *malEFGK* and the *msmsEFGK* operons lack the ATP-binding domain (*malK* and *msmK*), *A*. *schaalii* genome contains two paralogues of the *msiK* gene which encode for the ATP-binding components MsiK (G444DRAFT_01432 and G444DRAFT_01700) which shares 60% identity with MsiK from *Streptomyces* species. Previous studies showed that MsiK function as a universal ATPase that assists sveral ABC transporters [40]. The third CUT1 transporter consists of three open reading frames encoding for components of a putative multiple sugar transporter (G444DRAFT_01772 to G444DRAFT_01774). It is not known if this transporter has specificity for a particular carbohydrate. The substrate binding protein (G444DRAFT_01774) of this transporter shares 33% and 26% identities with homologues spr1534, Rv2041c and SCO6601 from *Streptococcus pneumoniae* R6, *Mycobacterium tuberculosis* H37Rv and *Streptomyces coelicolor* A3 [2], respectively, which are annotated as periplasmic-binding component of ABC transport systems specific for trehalose/maltose and similar oligosaccharides. However, the genes (*G444DRAFT_01772*, *G444DRAFT_01773* and *G444DRAFT_01774*) lie upstream of the *gal* operon, which encodes enzymes of the Leloir pathway for galactose metabolism (S3 Fig), and immediately downstream of the *aga* gene encoding for α-galactosidase GalA (G444DRAFT_01775), suggesting that *A*. *schaalii* ABC transporter is likely transports α-galactosides and or other galactose-containing oligosaccharides.

The three CUT2 (TC 3.A.1.2.-) transporters present in *A*. *schaalii* include homologues of the ribose ABC transporter RbsBAC (S4 Fig) and two transporters [(G444DRAFT-01074 to G444DRAFT_01076) and (G444DRAFT_00668 to G444DRAFT_00671)] annotated as multiple sugar transport and simple sugar transport systems, respectively. The CUT2 permease and substrate-binding proteins encoded by the genes (*G444DRAFT_01074* to *G444DRAFT_01076*; corresponding to *cheV*, *gguA*, *gguB*) are found associated with the *xylBAF* genes whose function related to the catabolism of xylose suggesting a possible candidate for a xylose ABC transporter (S5 Fig). The third CUT2 permeases and substrate-binding proteins encoded by the genes (*G444DRAFT_00668* to *G444DRAFT_00671*) are found associated with the *araBDA* genes whose functions related to the catabolism of L-arabinose suggesting a possible candidate for L-arabinose ABC transporter (S6 Fig).

Secondary transporters of the major facilitator (MFS) superfamilies catalyze the active uptake of solutes in reponse to chemiosmotic gradients [41]. MFS transporters are integral to membrane and usually consist of single polypeptide chain. Among the MFS transporters recognized in *A*. *schaalii* are those belonging to the Sugar Porter (SP) family (TC 2.A.1.1), the Organophosphate:Pi antiporter (OPA) family (TC 2.A.1.4), the Glycoside-Pentoside-Hexuronide:cation symporter (GPH) family (TC 2.A.2) and the Oxalate-Formate antiporter (OFA) family (TC 2.A.1.11). Four genes annotated as SP transporters were identified, three of which (*G444DRAFT_00662*, *G444DRAFT_00703* and *G444DRAFT_01089*) encode proteins possessing the Sugar_tr (pfam00083) domain and the remaining one (*G444DRAFT_01316*) encodes a protein possessing the Sugar_tr (pfam0790) domain. The gene (*G444DRAFT_00662*), which is located divergently oriented upstream of the *araRABD* operon (S6 Fig), encodes for L-arabinose permease AraE (G444DRAFT_00662). This pbservation suggests that *A*. *schaalii* contains two kinetically distinguishable systems for L-arabinose import: the AraE L-arabinose:H+ symporter (MFS transporter) and the ATP-driven system encoded by the previously mentioned genes (*G444DRAFT_00668* to *G444DRAFT_00671*).

The two genes (*G444DRAFT_00703*) and (*G444DRAFT_01089*) encode sugar porters which display 32% and 41% - 44% identities with the glucose transporter GlcP (SCO5578) from *Streptomyces coelicolor* A3 [2] and (sll0771) from *Synechocystis* sp. PCC 6803, respectively. The gene (*G444DRAFT_01316*), which is annotated as minor *myo*-inositol:H+ symporter (IolF), clustered with the rhamnose utilization genes *rhaDBAMR* (S7 Fig) and it therefore appears likely to function as L-rhamnose-proton symporter (RhaT). *A*. *schaalii* genome also contains two paralogous genes encoding for *sn*-glycerol-3-phosphate transporter GlpT (G444DRAFT_00967-G444DRAFT_00968), a member of the OPA family. GlpT mediates the translocation of glycerol-3-phosphate, which serves both as a carbon and energy source and a precursor for phospholipid biosynthesis. Furtheremore, *A*. *schaalii* has one *togT* gene encodes a protein (G444DRAFT_00165) homologous to TogT (TC 2.A.2.5.1), a member of the GPH family transporter, which mediates the uptake of oligogalagtorunides. Moreover, *A*. *schaalii* genome contains two paralogues (*G444DRAFT_00245* and *G444DRAFT_01505*) encoding for oxalate/formate antiporter (OxlT), a member of the OFA family proteins. OxlT function as an anion exchange carrier during the one-for-one antiport of divalent oxalate and monovalent formate [42]. This observation points to a dependency of *A*. *schaalii* on organic acids as sources of carbon.

In addition, *A*. *schaalii* contains members of the GntP family that include gluconate permeases similar to GntP from *E*. *coli* and *Bacillus* species. Five *gntP* paralogues were identified. All five genes encode proteins (G444DRAFT_00061, G444DRAFT_01083, G444DRAFT_01119, G444DRAFT_01676 and G444DRAFT_01823) annotated as Gluconate:H+ symporter (GNTP) and have pfam02447 domain. One of them (G444DRAFT_00061) accommodates fructuronate permease (TC 2A.8.1.3.) and one transporter (G444DRAFT_01676) accommodates high-affinity gluconate permease GntT (TC 2.A.8.1.4). Gluconate permeases were shown to be involved in gluconate uptake [43]. The GntP family is member of the ion transport (IT) superfamily [44].

#### Central carbohydrate metabolic pathways

*A*. *schaalii* has several features of anaerobic bacteria, as predicted from the genome sequence. The organism can potentially metabolize glucose to triose via three different pathways: gylcolysis [the Embden-Meyerhof (EMP) pathway], the Entner-Duodoroff (ED) pathway and the pentose phosphate pathway (S8 Fig). *A*. *schaalii* genome contains all genes encoding the enzymes necessary for the oxidation of carbohydrates to pyruvate via the EMP pathway. The genes coding for the key enzymes, fructose-1,6-biphosphatase (FBP, EC 3.1.3.11) and phosphoenolpyruvate carboxykinase (PEPCK, EC 4.1.1.49), necessary to direct carbon through gluconeogenesis are absent in the genome, suggesting that *A*. *schaalii* cannot perform gluconeogenesis. The oxidative branch of the pentose-phosphate pathway (OPPP) seems to be incomplete since the *pgl* gene encoding 6-phosphogluconolactonase 6PGL (EC 3.1.1.31) was missing from the genome. In contrast, all orthologous genes encoding enzymes for all steps of the nonoxidative branch of the pentose-phosphate pathway were present in *A*. *schaalii* genome.

**Fig 3:**
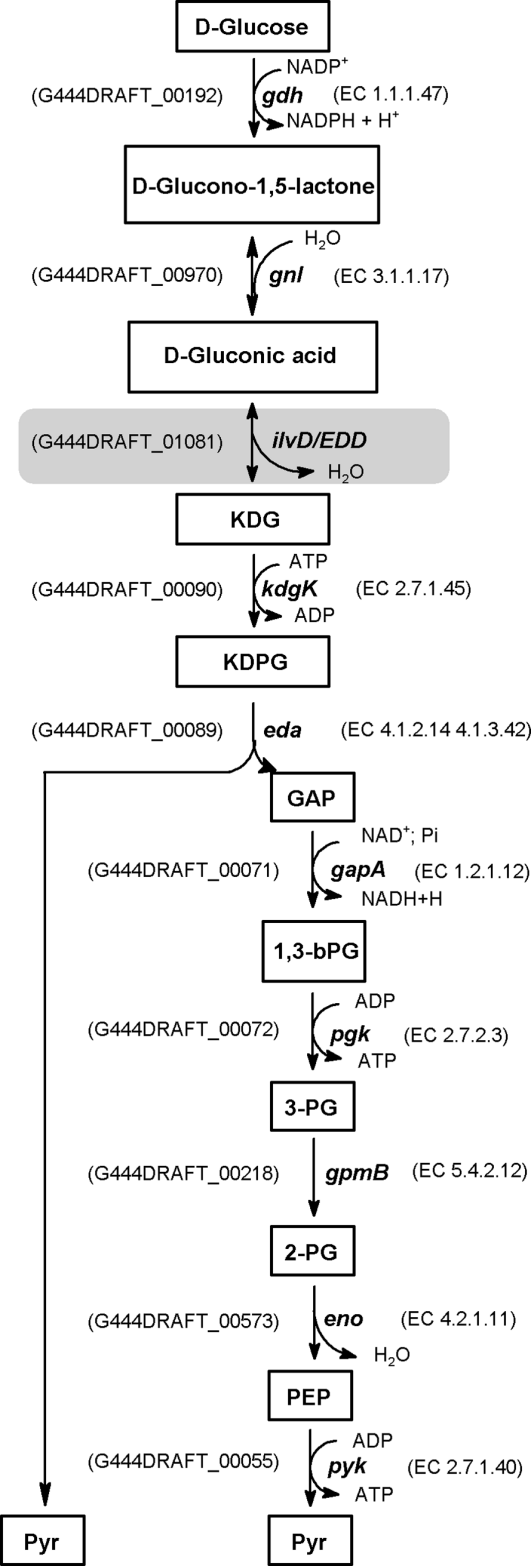
Semiphosphorylative Entner-Duodoroff pathway as it operates in *A*. *schaalii*. This pathway involves either oxidation of glucose by the membrane-bound glucose dehydrogenase (gdh) to form glucono-1,5-lactone which is then converted to gluconate by gluconolactonase or gluconate is taken up by the cell via a putative gluconate permease (GntP). Gluconate is then converted to 2-keto-3-deoxygluconate (KDG) by a specific gluconate dehydratase (ILVD_EDD). Further metabolism of KDG involves its phosphorylation by KDG kinase to form KDPG, followed by cleavage by EDA to pyruvate and glyceraldehyde-3-phosphate. Glyceraldehyde-3-phosphate is further converted to form another pyruvate molecule via common reaction of the EM pathway. A similar modified ED pathway has been shown to occur in several *Clostridium* species e.g. *Clostridium aceticum* [49] and halophilic archaea, e.g. *Halobacterium saccharovorum* [50]. Abbreviations: gdh, glucose-1-dehydrogenase; gnl, gluconolactonase; ilvD/EDD, dihydroxyacid dehydratase; KDGK, 2-dehydro-3-deoxygluconokinase; EDA, 2-dehydro-3-deoxyphosphogluconate aldolase; GAP, glyceraldehyde 3-phosphate dehydrogenase; PGM, phosphoglycerate mutase; ENO, enolase; PYK, pyruvate kinase.

Search for genes encoding the key enzymes of the ED pathway revealed the presence of the *eda* gene encoding 2-keto-3-deoxygluconate-6-phosphate aldolase (EDA) but lacks of the *edd* gene encoding 6-phosphogluconate dehydratase (EDD), thus disabling the ED pathway. Close inspection of the genome, however, revealed the presence of two genes, *ilvD* and (*G444DRAFT_01081*), predicted to encode dihydroxyacid dehydratase (DHAD) (G444DRAFT_01605; EC 4.2.1.9) and a putative hydratase (G444DRAFT_01081) of the YjhG/YagF family, repectively. Both dehydratases belong to the ILVD_EDD protein superfamily (pfam00920), members of which have been shown to be evolutionary related [45, 46]. Moreover, Kim and Lee [47] reported that DHAD exhibits substrate promiscuity with a high activity toward D-gluconate and some other pentonic and hexonic sugar acids and can catalyze the conversion of these sugar acids to 2-keto-3-deoxy analogs through a similar dehydration reaction. Therefore, we assume that the putative DHAD (G444DRAFT_01081), which is located adjacent to a gluconate:H+ symporter (G444DRAFT_01083), will compensate for the missing EDD enzyme in *A*. *schaalii*. Furthermore, analysis of genomic data revealed the presence of one copy of the *gnl* gene encoding gluconolactonase (EC 3.1.1.17), which catalyzes the conversion of gluconolactone to gluconic acid, and three copies of *kdgK* gene encoding 2-dehydro-3-deoxygluconokinase (EC 2.7.1.45), which catalyzes the phosphorylation of 2-keto-3-deoxygluconate (KDG) to 2-keto-3-deoxy-6-phosphogluconate (KDPG). The presence of the *eda*, (*G444DRAFT_01081*), *gnl*, *kdgK* genes in addition to *gdh* provides convincing evidence for the operation of a semiphosphorylative ED pathway in *A*. *schaalii*. Semiphosphorylative ED pathway was reported in halophilic archaea [48]. The schematic reactions of this pathway from glucose to pyruvate are presented in (Fig 3). Further experimental approaches with isotopic labeled tracers like ^13^C-labeled carbon sources are needed to elucidate pathway operation in *A*. *schaalii*.

The genes encoding for all enzymes necessary for the TCA cycle to operate in the reductive and the oxidative modes are absent in *A*. *schaalii*, except for the *fumC*, *sucB* and *lpdA* genes encoding fumarase (EC 4.2.1.2), the dihydrolipoamide succinyltransferase of the 2-oxoglutarate dehydrogenase component E2 (EC 2.3.1.61) and dihydrolipoamide dehydrogenase of 2-oxoglutarate dehydrogenase (EC 1.8.1.4), respectively. Neither of the genes coding for isocitrate lyase (EC 4.1.3.1) and malate synthase (EC 2.3.3.9) associated with operation of the glyoxylate bypass were present in the genome.

#### Fate of pyruvate

**Fig 4:**
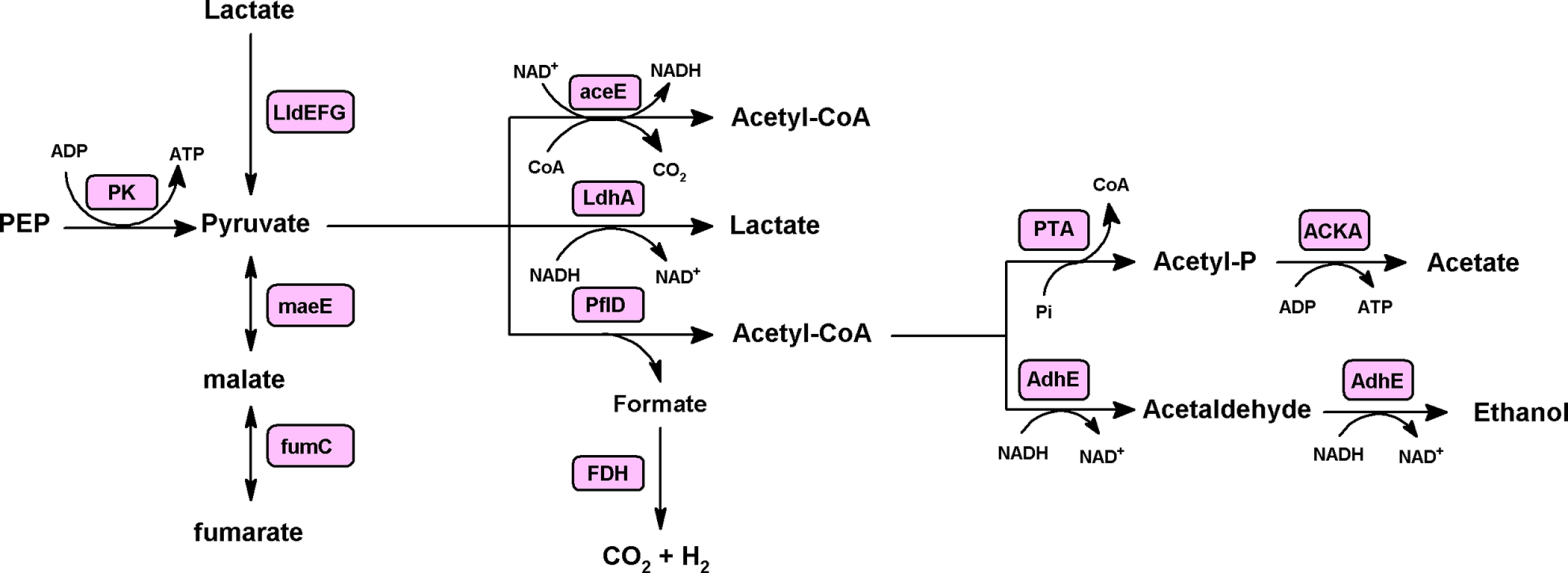
Scheme showing predicted pyruvate fermentation pathway in *A*. *schaalii*. The genes found in *A*. *schaalii* suggest that pyruvate is either oxidatively decarboxylated to acetyl-CoA via pyruvate dehydrogenase (pdh) or it may be reduced to lactate by the action of lactate dehydrogenase or it may be converted to acetyl-CoA and formate by pyruvate-formate lyase. Acetyl-CoA may be converted into ethanol, during which 2 NADH are oxidized via a fused acetaldehyde/alcohol dehydrogenase encoded by *adhE*, ot it may be converted to acetate through an acetyl phosphate intermediate using phosphotransacetylase (pta) and acetate kinase (ack) with the concomitant production of ATP. Abbreviations: LDH, L-lactate dehydrogenase; PTA, phosphotransacetylase; AK, acetate kinase.

Pyruvate generated by the EMP and the ED pathways may be fermented to lactate, acetate, formate and ethanol. The genes found in *A*. *schaalii* genome suggest a pathway for pyruvate fermentation as shown in (Fig 4). In this predicted pathway, pyruvate is oxidatively decarboxylated by pyruvate dehydrogenase PDH (EC 1.2.4.1) and/or pyruvate formate lyase PFL (EC 2.3.1.54), resulting in the formation of acetyl-CoA and CO_2_ or formate, respectively. Acetyl-CoA is then either converted to acetaldehyde by the action of acetaldehyde dehydrogenase domain of AdhE (EC 1.2.1.10 1.1.1.1) which is then reduced to ethanol by the action of alcohol dehydrogenase domain of AdhE (EC 1.2.1.10 1.1.1.1), or alternatively acetyl-CoA is converted to acetate with the generation of ATP in a two-stage reaction catalyzed by phosphate acetyltransferase PTA (EC 2.3.1.8) and acetate kinase AK (EC 2.7.2.1). Formate is converted to H_2_ and CO_2_ by the action of formate dehydrogenase FDH (EC 1.2.1.2). In agreement with the predicted pathway we identified the following putative genes and its enzyme products: two copies of the *pflD* gene encoding for pyruvate-formate lyase PFL (G444DRAFT_00375 and G444DRAFT_01725), *pta* gene encoding for phosphate acetyltransferase PTA (G444DRAFT_00377), *ackA* gene encoding for acetate kinase AK (G444DRAFT_00378), *adhE* gene encoding for the bifunctional acetaldehyde-CoA/alcohol dehydrogenase AdhE (G444DRAFT_01460) and *fdh* gene encoding for formate dehydrogenase (G444DRAFT_01038). The predicted pathway should be experimentally elucidated e.g., by using targeted mutagenesis.

Finally, the conversion of pyruvate to lactate is coupled to NADH oxidation and is catalyzed by lactate dehydrogenase LDH (EC 1.1.1.27) encoded by the *ldh* gene (G444DRAFT_01384). In addition, we identified a gene cluster (*G444DRAFT_01666* to *G444DRAFT_01669*) encoding components of lactate utilization machinary. This include an *lctP* gene encoding lactate permease (G444DRAFT_01666) and three genes *lldEFG* encoding components of a predicted L-lactate dehydrogenase complex LldEFG (G444DRAFT_01667, G444DRAFT_01668 and G444DRAFT_01669). These data suggest that *A*. *schaalii* can use L-lactate as a sole source of carbon and energy.

#### Fermentation and enegy conservation

Genome sequence analysis indicated that *A*. *schaalii* is incapable of respiratory metabolism, either aerobically or anaerobically. Except genes encoding for cytochrome *bd*-type quinol oxidase and genes encoding for the subunits of the proton driven F0F1-ATPase, we did not found the genes required for a complete electron transport chain that might be associated with aerobic or anaerobic respiration. The genome harbors the *cyd* operon encoding proteins required for the production of intact cytochrome *bd*-type quinol oxidase. The *cyd* operon consists of four genes: *cydAB* genes encode the structural proteins CydA (G444DRAFT_00230) and CydB (G444DRAFT_00231) and the *cydCD* genes encode an ABC transporter, CydD (G444DRAFT_00232) and CydC (G444DRAFT_00233), essential for the assembly of cytochrome *bd*. The expression of the *cydAB* operon is controlled by the two global transcriptional regulators ArcA/ArcB (G444DRAFT_00487/G444DRAFT_00486) and FNR (G444DRAFT_01491) encoded by the *arcA*, *arcB* and *fnr* genes, respectively [51]. Although anaerobic bacteria have no need of respiratory cytochrome oxidase, a functional cytochrome *bd* was found in e.g., *Bacteroides fraglis*, *Desulfovibrio gigas* and *Moorella thermoacetica* [52, 53, 54]. Cytochrome *bd* generates a PMF by transmembrane charge separation, but does so without being a “proton pump” [55]. Apart from PMF generation, cytochrome *bd* endows bacteria with a number of important physiological functions. Cytochrome *bd* facilitates both pathogenic and commensal bacteria to colonize O_2_-poor environments [52], serves as O_2_ scavenger to inhibit degradation of O_2_-sensitive enzymes [56, 57]. The *bd*-type cytochrome with high oxygen affinity is able to scavenge oxygen to such extent that it provides resistance against oxidative stress [58].

Another essential component of the respiratory chains is menaquinones, which delivers electrons and protons between dehydrogenases and cytochromes. Genomic data concerning the biosynthesis of menaquinones (MK) in *A*. *schaalii* are controversial. Despite clear chemotaxonomic evidence for the absence of menaquinones in all members of the genus *Actinotignum* including *A*. *schaalii* [1], bioinformatic analyses revealed the presence of a complete menaquinone biosynthesis pathway in the genome of *A*. *schaalii* DSM 15441^T^, enabled by a pentacistronic operon *menBCDEF* (G444DRAFT_00407 to G444DRAFT_00411) and three separately located genes, *menA* (G444DRAFT_00984), *ubiE* (G444DRAFT_00986) and a possible *menH* (G444DRAFT_01490). Paradoxically, however, lacks of *menDEF* and *menBCDEF* orthologs in the genome sequences of *A*. *schaalii* CCUG 27420^T^ and *A*. *schaalii* strain FB123-CAN-2, respectively, indicates an incomplete menaquinone biosynthesis pathway in these two strains.

Given the lack of: a TCA cycle, subunits of the reduced form of NADH dehydrogenase, and most other electron-transport chain complexes including menaquinone, we infer a strictly anaerobic fermentation-based lifestyle. *A*. *schaalii* is predicted to produce lactate, acetate and ethanol as fermentation end products. It catabolizes glucose via the EMP pathway to pyruvate and converts part of pyruvate to lactate by lactate dehydrogenase, thereby reoxidizing NADH produced during glycolysis to NAD^+^, while the other pyruvate part is cleaved to acetyl-CoA and formate by pyruvate-formate lyase (PFL). It cleaved half of the produced acetyl-CoA to acetate via acetylphosphate by two enzymes (acetate kinase and phosphate acetyltransferase) generating ATP by substrate-level phosphorylation (SLP), while the second half is reduced in two steps to ethanol with the oxidation of two NADH molecules to NAD^+^. The overall energy yield is three molecules of ATP per glucose molecule. This ATP is used for the synthesis of cellular macromolecules and other energy requiring processes in the cell and for the generation and maintenance of a proton-motive force (PMF) by the membrane bound F0F1-ATPase. The genome of *A*. *schaalii* contains an *atp* operon encoding the subunits of the proton driven F0F1-ATPase. This operon consists of eight genes (*G444DRAFT_01552* to *G444DRAFT_01559*) encoding components of the membrane-intrinsic F0 proton-channeling part (a, b, c subunits) and the membrane-extrinsic F1 catalytic part (α, β, γ, δ, ε). The membrane bound H^+^ F0F1-ATPase serves as a major regulator of intracellular pH by extruding protons from the cell at the expence of ATP [59, 60]. However, the physiological role of the H^+^ F0F1-ATPase in *A*. *schaalii* should be established by genetic and biochemicals means.

#### Correlations between genotype and phenotype

As previously mentioned, *A*. *schaalii* is capable of utilizing a wide range of carbon sources, including pentose sugar (arabinose, ribose and xylose), hexose sugars (glucose), and disaccharides (maltose and sucrose). Corresponding genes of these features could be found not only in the genome of *A*. *schaalii* DSM 15541^T^ but also in the genome of *A*. *schaalii* CCUG 27420^T^ and *A*. *schaalii* FB123-CAN-2.

L-arabinose metabolism in *A*. *schaalii* is achieved by three L-arabinose-catabolizing genes, *araA*, *araB* and *araD*, which comprised the *araBDA* operon. These genes encode three intracellular enzymes for arabinose catabolism, arabinose isomerase, ribulokinase and ribulose-5-phosphate epimerase, respectively. Upstream of the *araBDA* operon were the *araR* and *araE* genes, which were present in the opposite direction (S6 Fig). The *araE* gene encodes a proton symporter involved in the transport of arabinose into the cell.

The genes responsible for transport and utilization of D-ribose cluster together to form the *rbsBACR* operon. The *rbsBAC* genes encoding for the RbsB, RbsA and RbsCsubunits of the ribose ABC transporter (S4 Fig).

Xylose utilization in *A*. *schaalii* is mediated by the xylose catabolic enzymes, xylose isomerase and xylulose kinase, encoded by the *xylA* and *xylB* genes, respectively, of the *xyl* operon (S5 Fig).

*A*. *schaalii* possesses a PTS-independent pathway for glucose utilization. Glucose is imported via a non-PTS permease and phophorylated by glucokinase (Glk). Uptake and catabolism of the disaccharide maltose is mediated by the maltose/maltodextrin ABC transporter *malEFG* (S1 Fig). Genotype to phenotype correlations indicate that sucrose is metabolized *via* a non-PTS system, which consists of a sucrose hydrolase or invertase enzyme SacA (EC 3.2.1.26), a fructokinase ScrK (EC 2.7.1.4) and an as jet unidentified permease.

In addition, other genes associated with carbohydrate metabolism were found in the genome: (i) a *rhaADB* operon and *rhaM* gene whose annotation suggests that they encodes all enzymes involved in rhamnose mtabolim (S7 Fig); (ii) a *galRKTEM* operon encoding enzymes responsible for galactose catabolism (S3 Fig) and (iii) the *lldEFG* genes involved in D- and L-Lactate metabolism. These indicated that *A*. *schaalii* strains would have the potential abilities to metabolize rhamnose, galactose and lactate. However, the gene-phenotype correlation should be experimentally validated.

### Lipid metabolism

*A*. *schaalii* is able to synthesize fatty acids as well as other major lipid classes such as phospholipids, isoprenoids and glycolipids.

#### Fatty acids (FAs) biosynthesis

Analysis of the cellular FA profiles showed that strains of *Actinotignum schaalii* contain predominantly saturated and mono unsaturated straight chain FAs [1]. These include C_18:1_ω9*c* (> 50.0% of total FA content), C_16:0_ (> 13.0%) and C_18:0_ (> 4.0%). Bioinformatic analysis of *A*. *schaalii* genome revealed the presence of a single 9.09 kb gene (*fas*) encoding for Type I fatty acid synthase (FAS-I) [EC:2.3.1.-] (G444DRAFT_00340). FAS-I is a single, multifunctional polypeptide (320.58 kDa) that possesses all the catalytic sites required for *de novo* fatty acid biosynthesis.

#### Fatty acids degradation

The degradation of fatty acids to single acetyl-CoA units through the fatty acid β-oxidation cycle involves five enzymes: acyl-CoA synthetase, acyl-CoA dehydrogenase, enoyl-CoA hydratase, 3-hydroxyacyl-CoA dehydrogenase and 3-ketoacyl-CoA thiolase. Inspection of *A*. *schaalii* genome revealed the presence of three copies of *fadD* gene encoding long-chain fatty acid CoA synthetase ACSL (G444DRAFT_00265, G444DRAFT_ 00733 and G444DRAFT_00825; [EC 6.2.1.3]), one copy of a gene (*G444DRAFT_01439*) encoding acyl-CoA dehydrogenase (ACAD) and a copy of *atoB* gene encoding acetyl-CoA C-acetyltransferase (G444DRAFT_01613; [EC 2.3.1.9]). However, exhaustive bioinformatic search of the genome failed to identify genes encoding for enoyl-CoA hydratase and 3-hydroxyacyl-CoA dehydrogenase, indicating incomplete β-oxidation cycle. These genomic data suggest that *A*. *schaalii* cannot use exogenously supplied fatty acids as energy sources. This suggestion should be confirmed experimentally by e.g., enzymatic assays.

#### Phospholipids biosynthesis

The most abundant phospholipids found in the cell membrane of *A*. *schaalii* comprise cardiolipin (CL), phosphatidylgylcerol (PG), phosphatidylinositol (PI) and phosphatidylinositol monomannoside (PIM1); this composition is similar to that found in many other gram-positive bacteria. In addition, an unkown choline containing phosphoglycolipid (AbGL) has been identified [1]. These data are in line with protein data predicted from genome sequence analysis.

All the genes (except the *plsY* gene) encoding enzymes necessary for *de novo* biosynthesis of phospholipids were identified in *A*. *schaalii* genome. Phosphatidic acid (PA), the key phospholipid synthetic intermediate in prokaryotes, is generated from *sn*-glycerol-3-phosphate (G3P) by the consecutive acylation of *sn*-1 carbon followed by the *sn*-2 carbon of G3P, reactions catalyzed by the PlsX/PlsY and PlsC acyltransferases, respectively. Bioinformatics analysis showed that *A*. *schaalii* has two *plsC* genes encoding for 1-acyl-*sn*-glycerol-3-phosphate acyltransferase PlsC (G444DRAFT_00646 and G444DRAFT_01258; [EC 2.3.1.51]). Neither the *plsX* nor the *plsY* genes encoding for PlsX and PlsY homologues, respectively, were found in the genome. This raises the intriguing possibility that an as yet unidentified enzyme participates in PA biosynthesis may be present in *A*. *schaalii*.

PA is activated by CTP to form CDP-diacylglycerol (CDP-DAG) and the final polar head groups were added by replacing the CDP group. Bioinformatics explorations revealed the presence of the *cdsA*, *pgsA*, *cls* and *pgsA* genes encoding for homologues of phosphatidate cytidyltransferase CDS (G444DRAFT_00789) responsible for the synthesis of CDP-DAG, phosphatidylglycerol-3-phosphate synthase PGS1 (G444DRAFT_00812) catalyzes the synthesis of PG, cardiolipin synthase (G444DRAFT_01785) catalyzes the synthesis of CL from two molecules of PG and PI synthetase PIS (G444DRAFT_01210) catalyzes the synthesis of PI, respectively. *myo*-Inositol, the precursors of PI, is generated in *A*. *schaalii* by cyclization of glucose-6-phosphate to *myo*-inositol 1-phosphate via *myo*-inositol-1-phosphate synthase MIPS (G444DRAFT_00289; [EC 5.5.1.4]) encoded by *INO1* gene and subsequent dephosphorylation of *myo*-inositol 1-phosphate to *myo*-inositol via histidinol–phosphate phosphatase HisN (G444DRAFT_01519; EC 3.1.3.15) encoded by *hisN* gene. HisN belongs to the inositol-monophosphatase (IMP) family (pfam00459) and showed high sequence identity to inositol-1-monophosphatse (EC 3.1.3.25), that hydrolyse the ester bond of *myo*-inositol-1-phosphate.

Furthermore, genes encoding enzymes involved in the mannosylation of the inositol residue of PI for the synthesis of glycolipids (PIMs) were identified in the genome. These include genes predicted to encode enzymes responsible for synthesis of GDP-mannose, the activated form of mannose, required for mannosylation processes and genes encoding enzymes predicted to encode glycosyltransferases required to incorporate the activated mannose into PI. Two of the three key genes, *manA*, *manB* and *manC*, responsible for GDP-mannose synthesis are present in the genome: *manA* encoding mannose-6-phosphate isomerase PMI (G444DRAFT_00639; [EC 5.3.1.8]) that catalyzes the conversion of fructose-6-phosphate to mannose-6-phosphate and *manC* encoding for mannose-1-phosphate guanylyltransferase GMPP (G444DRAFT_00325; [EC 2.7.7.13]) which catalyzes the synthesis of GDP-mannose from mannose-1-phosphate and GTP. The *manB* gene encoding phosphomannomutase PMM (EC 5.4.2.8) that catalyzes the interconversion of mannose-6-phosphate to mannose-1-phosphate is absent in *A*. *schaalii* genome. It is likely that the PMM activity may be contributed by an additional phosphohexomutase such as phosphoglucomutase PGM (G444DRAFT_01405; [EC 5.4.2.2]) and/or phosphoglucosamine mutase GlmM ([G444DRAFT_00418; EC 5.4.2.10]), which contain the four domains required to catalyze the transfer of the phosphate group between C6 and C1 positions of glucose-6-phosphate and glucosamine-6-phosphate, respectively. This explanation need to be verified by experimental studies.

The genome also harbors the *pimA* gene encoding phosphatidylinositol α-mannosyltransferase PimA (G444DRAFT_01212; [EC 2.4.1.57]), which catalyzes the transfer of a mannose residue from GDP-mannose to the 2-position of the *myo*-inositol ring of PI leading to the synthesis of phosphatidylinositol monomannoside (PIM1). Bioinformatic search of annotated genome failed to reveal the existence of *pimB* gene encoding phosphatidylinositol α-1,6-mannosyltransferase responsible for the transfer of a mannose residues from GDP-mannose to 6-position of the *myo*-inositol ring of PIM1. This finding is consistent with the absence of PIM2 in the cell wall of *A*. *schaalii*.

#### Isoprene biosynthesis

Inspection of the genome revealed that *A*. *schaalii* possesses a complete mevalonate (MVA) pathway for isoprene biosynthesis. On the other hand, genes coding for the enzymes catalyzing the alternative MEP pathway are not found in the genome. The MVA pathway utilizes acetyl-CoA as the primary precursor for the production of the two main isoprenoid precursors, namely isopentenyl diphosphate (IPP) and dimethylallyl diphosphate (DMAPP). Two acetyl-CoA molecules are condensed to form acetoacetyl-CoA by acetyl-CoA acetyl transferase (EC 2.3.1.9) encoded by *atoB* gene. Acetoacetyl-CoA is further condensed with acetyl-CoA to form 3-hydroxy-3-methylglutaryl CoA (HMG-CoA), a reaction brought about by hydroxymethyl-glutaryl-CoA synthase HMGS (EC 2.3.3.10) encoded by *hmgS* gene. Subsequently, four enzymes encoded by *hmgCR*, *mvaK1*, *mvaK2*, and *mvaD* genes catalyze sequential reactions converting acetyl-CoA to IPP which can be interconverted to its isomer DMAPP by isopentenyl pyrophosphate isomerase IDI (EC 5.3.3.2) encoded by *idi* (*fni*) gene. The genome also harbors the genes encoding for geranylgeranyl diphosphate synthase IdsA (EC 2.5.1.1 2.5.1.10 2.5.1.29), farnesyl diphosphate synthase UppS (EC 2.5.1.31) and heptaprenyl diphosphate synthase HepST (EC 2.5.1.30). IdsA (G444DRAFT_01177) catalyzes the condensation of DMAPP and IPP to produce geranyl pyrohosphate (GPP), farnesyl pyrophosphate (FPP) and larger order isoprenoid compounds. Both UPPS (G444DRAFT_00560) and HepST (G444DRAFT_00987) also catalyze the biosynthesis of larger order polyprenyl pyrophosphates.

### Protection against oxidative stress

*A*. *schaalii* is a gram-positive anaerobic bacterium that normally grows without oxygen (or in the presence of minimal concentrations of oxygen). Exposure of the organism to air can give rise to the metabolic conversion of atmospheric oxygen to reactive oxygen species (ROS), which pose significant threat to cellular integrity in terms of their damage to proteins, lipids, RNA and DNA. Like other anaerobic bacteria, *A*. *schaalii* has developed several mechanisms to protect itself from the damaging effects of ROS. Inspection of *A*. *schaalii* genome revealed the presence of 13 antioxidant-related genes encoding several antioxidant enzymes that confer resistance against toxicity of ROS including cytochrome *bd* oxidase, superoxide dismutase (SOD), peroxiredoxins (PRX) and thioredoxins (TRX).

#### Cytochrome *bd* (*cyd*)

As previously mentioned *A*. *schaalii* harbors the *cydAB* operon encoding a cytochrome *bd* oxidase. Cytochrome *bd* has high affinity for oxygen, making it an effective oxygen scavenger protecting bacterial cell against oxidative stress conditions [55, 61]. It prevents the formation of H_2_O_2_ and ROS by reducing molecular oxygen to H_2_O [62]. The presence of cytochrome *bd* in strict anaeorobes may desensitize such bacteria to certain levels of oxygen permitting growth in nanomolar concentration of oxygen [52].

#### Superoxide dismutase (SOD)

*A*. *schaalii* harbors *sodA* gene encoding for Fe/Mn-containing superoxide dismutase (G444DRAFT_01169), which contributes to aerotolerance in this bacterium. SOD catalyzes the dismutation of the superoxide radical (O_2_) to oxygen and H_2_O_2_, which can be further reduced to water and oxygen by catalase or peroxiredoxins. Since *A*. *schaalii* does not possess catalase, detoxification of H_2_O_2_ is accomplished by peroxiredoxins.

#### Peroxiredoxins (Prxs)

*A*. *schaalii* genome harbors *bcp* gene encoding for bacterioferritin comigrating protein BCP (G444DRAFT_00268). BCP is a member of the TSA/AhpC peroxiredoxin family. Prxs are cysteine-based peroxidases (EC 1.11.1.15) that use their highly reactive cysteine residues to reduce and detoxify H_2_O_2_, organic hydroperoxides and peroxynitrite converting them to water, alcohols or nitrite, respectively [63, 64].

In addition *A*. *schaalii* genome harbors *osmC* gene encoding a protein of 136 amino acids (G444DRAFT_01716) predicted as uncharacterized OsmC-related protein (COG1765). OsmC (osmotically inducible protein) possess thiol-dependent peroxidase activity and known to be involved in the cellular defense against oxidative stress caused by exposure to organic hyperoxides or elevated osmolarity [65, 66].

#### Thioredoxins (Trxs)

*A*. *schaalii* genome harbors two genes, *trxA* and *trxB*, encoding thioredoxin (G444DRAFT_00223) and thioredoxin reductase (G444DRAFT_01284; [EC 1.8.1.9]), repectively. Thioredoxins are kept in the reduced thiol state by thioredoxin reductase. Thioredoxins are low-molecular weight proteins with redox-active cysteine residue that play a key role in protection of cells against oxidative stress, either by reducing protein disulfide bonds produced by various oxidants or by scavenging ROS [67].

### Defense of genome integrity and cell fitness

Genome sequence analysis revealed that *A*. *schaalii* has developed various mechanisms that may allow it to withstand viral and alien nucleic acid invasion. These include postsegregation killing systems (also called addiction modules because their loss leads to the death of their host bacterium) and Clustered regularly interspaced short palindromic repeats (CRISPR) and CRISPR-associated (Cas) proteins (CRISPR-Cas systems). Two kinds of postsegregation killing systems are present in *A*. *schaalii* genome: toxin-antitoxin (TA) systems and restriction-modification (RM) systems.

#### Toxin-antitoxin (TA) systems

Toxin-antitoxin (TA) systems are small mobile genetic modules found on bacterial mobile genetic elements like plasmids as well as bacterial chromosomes [68]. They are generally composed of bicistronic operons that encode a stable toxin preceded by its cognate unstable antitoxin. Reverse organization, in which the toxin gene preceds that of the antitoxin within the operon, as well as three-component systems were reported [69]. Toxins are always small proteins (<130 amino acids), whereas antitoxins can be proteins or RNAs. Chromosomal TA systems were shown to be involved in numerous cellular processes related to programmed cell death [70], maintenance of mobile genetic elements [71], phage abortive infection [72], stress response [73], biofilm formation [74], virulence [75], persistence [76] and other cellular processes [77].

A thorough bioinformatic search of type II TA systems in genomes of *A*. *schaalii* strains led to the identification of ≈19 loci of TA genes, of which three pairs of genes encoding TA belonging to previously known TA systems (RelBE, HicAB and ζ). Four genes encoding antitoxins (RelB, HigA, HicB and Phd) that are not associated with cognate toxins suggesting that they might be solitary antitoxins. In addition, based on the “mix” and “match” principal which suggests that type II TA toxins can also interact with antitoxins from different classes [78], we identified twelve TA gene pairs encoding for twelve putative novel TA systems (Table 2). These candidate systems should be experimentally validated.

**Table 2:** Toxin-antitoxin systems of *Actinotignum schaalii*.

No.	TA Systems	Antitoxin	Antitoxin locus Tag	Length (aa)	COG	Toxin	Toxin locus Tag	Length (aa)	COG	Comments
**1**	relBE	RelB/DinJ	G444DRAFT_00903	50	—	ParE/RelE	G444DRAFT_00902	44	3668	
**2**	hicAB	HicB	G444DRAFT_01494	124	—	HicA	G444DRAFT_01493	61	—	
**3**	ζ					ζ	G444DRAFT_00631	271	—	
**4**	COG3636-ParE	HTH (Xre family)	G444DRAFT_00657	97	3636	ParE Gp49 (DUF891)	G444DRAFT_00656	100	3657	Toxin precedes antitoxin
**5**	MNT- HEPN	HEPN (DUF86)	G444DRAFT_01087	116	2361	MNT	G444DRAFT_01088	113	1669	
**6**	Xre-COG2856	HTH_31 (Xre family)	G444DRAFT_01409	112	1396	DUF 955 (Zn-dependent peptidase ImmA, M78 family	G444DRAFT_01408	256	2856	
**7**	MerR-COG2856	HTH_17 (MerR superfamily)	G444DRAFT_01241	150	—	DUF 955 (Zn-dependent peptidase ImmA, M78 family	G444DRAFT_01242	383	2856	
**8**	RelB-GNAT	RelB (RHH)	G444DRAFT_01680	94	3077	GNAT (pfam00583)	G444DRAFT_01682	186	2153	
**9**	Xre-X1	HTH_XRE (HipB)	G444DRAFT_00618	86	—	Hypothetical protein	G444DRAFT_00619	127	—	
**10**	HigA-COG1396	HTH_3 HigA	G444DRAFT_00678	99	—	HTH_3 Xre family	G444DRAFT_00679	113	1396	
**11**	Xre-DUF3046	HTH_31 (Xre family) HipB	G444DRAFT_00814	126	—	DUF3046	G444DRAFT_00815	78	—	
**12**	AbrB/MazE-Phd	Phd	G444DRAFT_00868	80	—	AbrB/MazE	G444DRAFT_00869	86	—	
**13**	X-Fic/Doc	hyp. protein	G444DRAFT_00925	178	—	Fic/Doc	G444DRAFT_00926	266	3177	
**14**	Xre-X2	HTH_3 (Xre family)	G444DRAFT_00977	75	—	Hypothetical protein	G444DRAFT_00978	79	—	
**15**	RelB-Spdl/YhfL	RelB	G444DRAFT_01366	98	3077	Sdpl/YhfL	G444DRAFT_01367	131	—	
**16**	Solitary antitoxin	RelB	G444DRAFT_00870	90	3077					
**17**	Solitary antitoxin	HTH_3 (HigA family)	G444DRAFT_00912	82	3093					
**18**	Solitary antitoxin	HicB	G444DRAFT_01803	72	1598					
**19**	Solitary antitoxin	Phd/YeFM_antitox	G444DRAFT_01805	86	2161					

#### Restriction modification systems (RM)

Restriction modification systems (RM) protect bacteria from infection by mobile genetic elements such as phages. RM systems consist of a methyltransferase (MTase) that modifies a specific DNA sequences in a genome by methylation and a restriction endonuclease (REase) that cleaves unmethylated DNA [79]. Searching genes encoding Mtase and REase revealed that *A*. *schaalii* predicted to harbor the genetic determinants for Type I, II and III RM systems.

Type I RM is encoded by two adjacent similarly oriented genes. One gene (*G444DRAFT_00292*) encoding a protein (356 amino acids) which contains two target recognition domains (TRDs) and is responsible for the recognition of target DNA sequence and the other gene (*G444DRAFT_00293*) encoding a methyltransferase (666 amino acids) that contains AdoMet binding motifs. BLASTP analysis revealed that the deduced amino acid sequence of the proteins (G444DRAFT_00292) and (G444DRAFT_00293) shares significant homology (45–77% identities) with previously characterized *BcgI*-RM system from various bacteria.

The genome harbors Type IIS RM system. This consists of two adjacent similarly oriented genes. The gene (*G444DRAFT_01011*) encoding a polypeptide (592 amino acids), which contained a putative conserved domain (pfam09491) related to the RE_AlwI superfamily of restriction endonucleases. The second gene, *dam* gene, located upstream of AlwI, encoding a DNA adenine methyltransferase (Dam) (G444DRAFT_01012), which showed a close similarity (32% identity) to homolog (b3387; M.EcoKDam MTase) of *E*. *coli*.

The putative Type III RM system is encoded by two distantly located genes. The gene (*G444DRAFT_00278*) encoding the restriction endonuclease subunit (Res) that contains DEAD-box motifs present in superfamily II DNA or RNA helicases. The second gene (*G444DRAFT_00547*) encoding putative N6 adenine-specific DNA methyltransferase (EC 2.1.1.72) with pfam01555-N6_N4_Mtase conserved domain.

#### CRISPRs

CRISPRs provide bacterial cells with an acquired resistance against invading genetic elements such as phages or plasmids [80]. CRISPR/*cas* systems composed of two specific determinants: (i) clustered regularly interspaced short palindromic regions (CRISPR array) and (ii) regions encoding CRISPR-associated (Cas) proteins. Genomic analysis revealed that *A*. *schaalii* genome possesses two CRISPR loci. CRISPR locus 1 contains 333bp, harbors 5 spacer sequences, the length of the cosensus sequence of the direct repeat is 29bp (GGAATTACCCCCGCTTGCGCGGGGAACAG). CRISPR locus 2 contains 1065bp, harbors 17 spacer sequences, the length of consensus sequence of the direct repeat is 29bp (GGAATTACCCCCGCTTGCGCGGGGAACAG). Eight CRISPR-associated (*cas*) genes were identified. These include: *cas2* (G444DRAFT_01617), *cas1* (G444DRAFT_01618), *cas3* (G444DRAFT_01619), *cas6* (G444DRAFT_01620), *cse1* (G444DRAFT_01621), *cse2* (G444DRAFT_01622), *cse4* (G444DRAFT_01623) and cas5 (G444DRAFT_01624)].

#### Phage abortive infection system (Abis)

Genome sequence analysis revealed the presence of an ORF encoding a phage-resistance protein (G444DRAFT_01685) with Abi_2 domain (pfam07751). Abis are post-infection resistance mechanisms that hinder the propagation of phages in bacterial populations by inducing death of infected cells. This decreases the number of progeny particles and limits their spread to other cells allowing the bacterial population to survive.

### Invasion-associated proteins (Virulence factors)

Genomic analysis revealed that *A*. *schaalii* expresses several virulence-associated factors that allow the organism to colonize and evade host tissues, including adhesin and fimbrial proteins, degradative enzymes such as sialidase and NlpC-P60 protein, heat-shock proteins and secretion of protein with WXG100 domain.

*A*. *schaalii* genome contains two clusters of tight adherence (*tad*) genes, which are located on different regions on the chromosome: *tadA* gene encoding ATPase of CpaF family TadA (G444DRAFT_00310 and G444DRAFT_01487); *tadB* gene encoding inner membrane protein TadB (G444DRAFT_00309 and G444DRAFT_01486); and *tadC* gene encoding inner membrane protein TadC (G444DRAFT_00308 and G444DRAFT_01485). The *tad* genes are organized linearly in the same direction, indicating that they consititute an operon. The *tad* genes encode the machinery required for the assembly of pili. The *tad* locus has been implicated in the pathogenesis of several bacterial diseases. Pili, which are encoded within pathogenicity islands, play major roles in adhesion and host colonization [81]. Currently there was no information about the functional significant of pili in *A*. *schaalii* and the functions of the *tad* loci in *A*. *schaalii* should be investigated in experimental study.

*A*. *schaalii* genome carries three genes predicted to encode sortase A (SrtA) enzymes. However, database search using PSI-BLAST analysis leaves no doubt that two gene products (G444DRAFT_00284 and G444DRAFT_00353) were homologues of sortase C (SrtC) and the third gene product (G444DRAFT_01330) was a homolog of sortase E (SrtE). The *srtC* genes, encoding SrtC, clustered in two separate loci (*G444DRAFT_00284*, *G444DRAFT_00285*, *G444DRAFT_00286* and *G444DRAFT_00353*, *G444DRAFT_000354*, *G444DRAFT_00355*) with gene encoding putative surface protein. The gene products (G444DRAFT_00285 and G444DRAFT_000354) were identified by PSI-BLAST analysis as fimbrial assembly proteins, whereas the gene products (G444DRAFT_00286) and (G444DRAFT_00355) were identified as Cna protein B-type domain and cell wall anchor protein with LPXTG-motif, respectively. This suggests that *A*. *schaalii* genome expresses two types of adhesive fimbriae. However, further studies are needed to explore the role of the C sortases in fimbrial biogensis and to examine their distribution on the cell surface of this organism.

Another virulence-associated gene found in *A*. *schaalii* genome was the *nanI* gene encoding an exo-alpha-sialidase (G444DRAFT_01683; [EC 3.2.1.18]). Sialidase contribute to the removal of terminal sialic acid from host tissues and cells and is considered a significant virulence factor in term of adhesion and immune modulation.

*A*. *schaalii* genome also harbors genes encoding heat shock proteins (Hsps). These include genes encoding IbpA (Hsp20; G444DRAFT_01634), GrpE (Hsp20; G444DRAFT_00895), DnaK (Hsp70; G444DRAFT_00894), DnaJ (Hsp40; G444DRAFT_00896), GroEL (Hsp60; G444DRAFT_00649), ClpB (Hsp100; G444DRAFT_00927) and ClpC (Hsp100; G444DRAFT_01067). Heat shock proteins are molecular chaperones that are induced in response to adverse environmental conditions such as elevated temperature. Besides its houskeeping activities, including promotion of correct folding of non-native proteins and prevention of proteins aggregation, they have potential roles in aiding pathogen virulence [82]. They are implicated as adhesins for invading the host cells and are potent immunogens and active immunomodulatores [83].

In addition to the above mentioned virulence genes, *A*. *schaalii* genome harbors genes that encode Esat-6 secretion system (ESX or Ess) also known as type VII secretion system (T7SS). The Esx-1 gene cluster in *A*. *schaalii* composed of the two-gene operon *esxA/esxB* and the two genes, *eccC* and *eccB*, located in close proximity of the *esxA/esxB* operon. The *esxA* and *esxB* genes encoding small secreted proteins EsxA (G444DRAFT_00919; 95 amino acids) and EsxB (G444DRAFT_00918; 111 amino acids), respectively, containing WXG100 motif. The *eccC* gene encodes a transmembrane protein of the FtsK-SpoIIIE ATPase family (G444DRAFT_00923) and the *eccB* encodes a protein with transmembrane domainB EccB (G444DRAFT_00921). A second *eccC* gene predicted to encode FtsK-SpoIIIE ATPase (G444DRAFT_00811) located elsewhere in the genome. Proteins of the Esx-1 system were identified in *Actinobacteria* such as *Mycobacterium tuberculosis* and *Corynebacterium diphtheriae* and members of the Firmicutes (low G+C Gram-positive bacteria) such as *Staphylococcus aureus*, *Streptococcus agalactiae* and *Listeria monocytogens*. The Esx-1 system plays an important role in the virulence of the human pathogens *M*. *tuberculosis* and *Staphylococcus aureus* [84, 85]. Therefore, we speculate that the Esx-1 system in *A*. *schaalii* may contribute to virulence of this organism. However, this speculation should be supported by experimental studies.

### Resistant to antibiotics

As previously mentioned, *in vitro* susceptibility showed that *A*. *schaalii* is resistant to ciprofloxacin and metronidazole.

**Fig 5:**
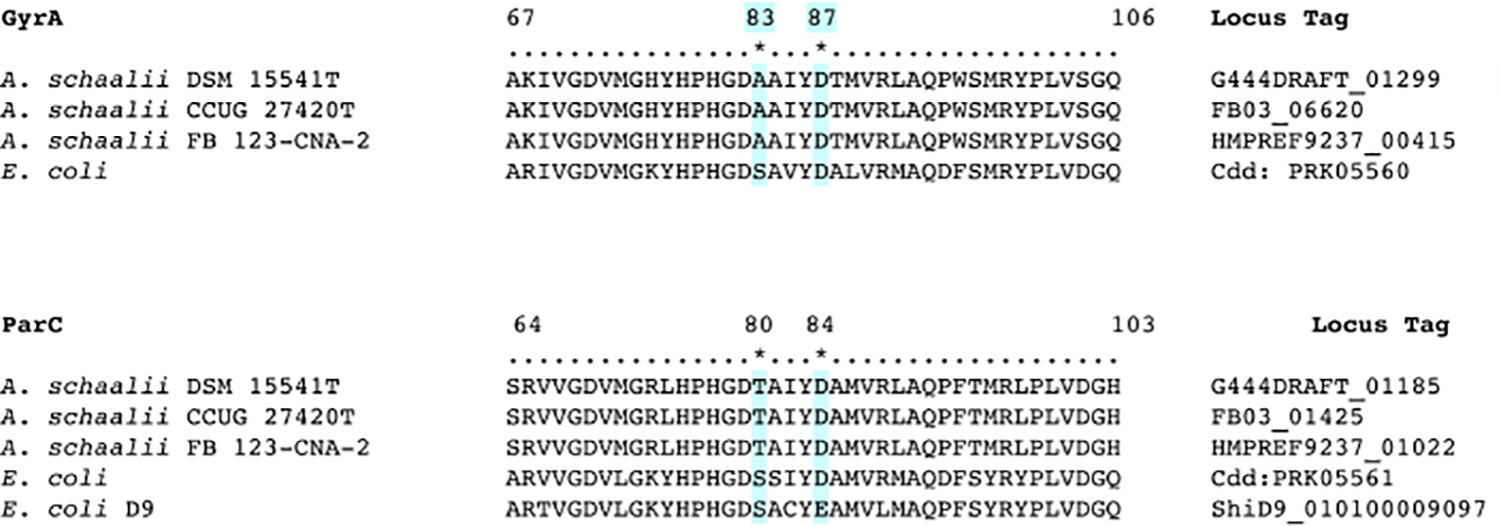
Comparison of the amino acid sequences of the QRDRs of both GyrA and ParC in *A*. *schaalii* with the equivalent region of *E*. *coli*. Hotspots for mutation giving rise to fluoroquinolone resistance are at serine 83 and aspartate 87 of the GyrA and at serine 80 and aspartate 84 of ParC (according to *E*. *coli* numbering).

Fluoroquinolones such as ciprofloxacin are known to exert their bactericidal activity by acting on the bacterial target enzymes DNA gyrase (GyrA) and topisomerase IV (ParC). Resistance occurs mainly as a result of single point mutation within the quinolone resistance-determining region (QRDR) of the *gyrA* and the *parC* genes [86, 87]. Examination of the QRDRs in the genome sequences of *A*. *schaalii* DSM 15541^T^, *A*. *schaalii* CCUG 27420^T^ and *A*. *schaalii* FB 123-CAN-2 revealed the presence of a single mutation at both *gyrA* (position 83 according to *Escherichia coli* numbering) and in *parC* (position 80) genes resulting in changes of Ser83→Ala and Ser80→Thr, respectively (Fig 5). This observation corresponds well with those of earlier studies [88].

The molecular mechanism that governs antimicrobial resistance to metronidazole is provided by the activity of nitroimidazole reductase. This enzyme is essential to convert metronidazole from a harmless prodrug to a bactericidal agent [89]. Specific resistance genes (*nim*) have been identified in several genera of Gram-positive and Gram-negative anaerobic bacteria such as *Peptostreptococcus* and *Bacteroides* species [90, 91]. In contrast to these bacteria, the genome sequences of *A*. *schaalii* DSM 15541^T^, *A*. *schaalii* CCUG 27420^T^ and *A*. *schaalii* FB 123-CAN-2 lack the *nim* genes, instead, the genome sequences of the three strains contain *frxA* genes (*G444DRAFT_01234*; *FB03*:*01660*; *HMPREF9237_01071*), which encode NADPH-flavin oxidoreductase. FrxA belongs to the nitroreductase protein family (pfam00881; KO:K00540) and catalyzes the reduction of nitrocompounds using NADPH as electron donor. Earlier studies showed that FrxA enhances metronidazole resistence in *Helicobacter pylori* [92]. Therefore, we assume that the *frxA* gene may be involved in metronidazole resistance among *A*. *schaalii* strains. However, further study will be required to determine what role the *frxA* gene plays in metronidazole resistance in *A*. *schaalii*.

## Conclusions

The draft genome sequence of *A*. *schaalii* provided numerous insights into the metabolic, physiologic and virulence potential of this organism. In general the predicted physiological capabilities are in good agreement with reported experimental observations. A complete glycolytic pathway is present leading to the production of pyruvate, which can subsequently be converted to lactate, acetate and ethanol. *A*. *schaalii* contains an intact nonoxidative branch of the pentose phosphate pathway, but lacks the oxidative branch due to absence of the gene encoding 6-phosphogluconolactonase. *A*. *schaalii* is predicted to possess a standard as well as a semiphosphorylative variant of the Entner-Doudoroff pathway. The TCA cycle and the glyoxylate shunt are completely absent. The organism lacks a functional gluconeogenesis due to absence of genes encoding for fructose-1,6-bisphosphatase and PEP carboxykinase. Energy generation is primarily dependent on substrate level phosphorylation in glycolysis and fermentation. *De novo* fatty acids biosynthesis is carried out by multifunctional type I fatty acid synthase. A full set of genes necessary for the biosynthesis of cardiolipin, phosphatidylglycerol, phosphatidylinositol and phosphatidylinositol monomannoside are present in the genome. For detoxification of reactive oxygen species (ROS) genes encoding several proteins such as superoxide dismutase, thioredoxin, thioredoxin reductase and Bcp were identified. For resistance against invading phage, the genome harbors gene encoding for type II toxin-antitoxin system, different RM systems, two CRISPR loci and a gene encoding for phage abortive system. *A*. *schaalii* genome harbors several virulence related genes, including genes encoding for sortase-associated pili, a gene encoding sialidase (NanI), genes encoding for secreted proteins with WXG100 domain (T7SS), genes encoding for Hsp proteins. The observed resistance to ciprofloxacin correlates with the presence of point mutation in the protein products of the *gyrA* and *parC* genes. The detailed knowledges presented here are based on an *in silico* approach and provides a template for future experimental analysis for its validation.

## Supporting information

S1 Fig Genomic organization of the maltose utilization genes in *A*. *schaalii* DSM 15541^T^. The maltose/maltodextrin ABC transporter encoded by the *malEFG* gene cluster. The organization of the locus within the (*G444DRAFT_01575*—*G444DRAFT_01576*—*G444DRAFT_01577*) gene cluster is similar to that observed in the genomes of other *Actinobacteria* e.g. *Streptomyces coelicolor* and *Streptomyces erythrea*. Abbreviations: *malE* encodes a maltose-binding protein; *malF* and *malG* encode permeases of the ABC transporter; *aglA* encodes α-glucosidase; *malR* encodes transcriptional regulator of the LacI family. Two copies of the *malK* genes encoding the ATPase are located elsewhere in the genome. Orthologs are shown by matching colors.
(TIF)

S2 Fig Genomic organization of the multiple sugar metabolism (msm) transporter in *A*. *schaalii* DSM 15541^T^. The multiple sugar ABC transporter contains *msmXEFGK* gene cluster. Abbreviations: *malZ* encodes α-amylase (EC 3.2.1.20); *msmE* encodes a sugar-binding protein; *msmF*, *msmG* encode two permeases; *msmK* encodes an ATP-binding protein located elsewhere in the genome.
(TIF)

S3 Fig Genomic organization of the multiple sugar ABC transporter and the *gal* operon in *A*. *schaalii* DSM 15541^T^. The galactose operon comprises the *galRKTEM* genes encoding a repressor and enzymes for the Leloir pathway for galactose metabolism. The *gal* opern clusters together with three genes encoding for a multiple sugar ABC transporter. Abbreviation: *galR*, *galK*, *galT*, *galE* and *galM* encode *gal* repressor (ROK family), galactokinase (EC 2.7.1.6), galactose-l-phosphate uridyltransferase (EC 2.7.7.12), UDP glucose-4-epimerase (EC 5.1.3.2) and aldose-1-epimerase (EC 5.1.3.3), respectively. The *galR* and *galK* are divergently oriented with respect to the *galT* and *galE* genes. The *galM* is not a part of the *gal* operon and was located elsewhere in the chromosome. On the opposite strand to *galR* and transcribed divergently are four open reading frames encoding for an ABC transporter of the CUT1 family (G444DRAFT_01772 to G444DRAFT_01774) and α-galactosidase (G444DRAFT_01775). Two importers, one in *E*. *coli* and the second in *M*. *tuberculosis*, presenting similar organization: the glycerol-3-phosphate transporter in *E*. *coli* encoded by genes (*ugpA*-*ugpE*-*ugpB*-*ugbC*) and the sugar transporter in *M*. *tuberculosis* encoded by genes (*sugA*-*sugB*-*sugC*-*lpqY*). *A*. *schaalii* transporter, however, differs from *E*. *coli* and *M*. *tuberculosis* in lacking homologs the *ugpC* and *sugC* genes, respectively. Arrows indicate direction of transcription.
(TIF)

S4 Fig Genomic organization of the D-ribose utilization genes in *A*. *schaalii* DSM 15541^T^. The *rbsBACR* gene cluster is responsible for the metabolism of ribose. The *rbsBAC* genes encoding for the ABC transporter belonging to the CUT2 family, where *rbsB* encodes a ribose-binding protein, *rbsA* encodes ATP-binding protein and *rbsC* encodes a permease. In addition to the *rbsA* gene, the genome harbors three *rbsK* genes encoding three ribokinases (EC 2.7.1.15), which specifically directs its phosphorylating activity towards d-ribose, converting this pentose sugar to ribose-5-phosphate. The transcription of the *rbs* gene cluster is regulated by a LacI-type regulator encoded by *rbsR*, located immediately upstream of *rbsB*.
(TIF)

S5 Fig Genomic organization of the xylose transporter and xylose utilization genes. The xylose ABC transporter genes (*cheV* and *gguAP*) cluster with the xylose utilization genes *xylBRA*. Abbreviations: *cheV* encodes a xylose-binding protein; *gguA* encodes ATP-binding protein; *gguP* encodes permease of the ABC transporter; *xylA* encodes xylose isomerase (EC 5.3.1.5); *xylB* encodes xylulokinase (EC 2.7.1.17); *xylR* encodes transcriptional regulator of the ROK /IclR family. The products of the *xylA* and *xylB* genes together catalyze the conversion of xylose to xylulose-5-phosphate. The location of the *xylR* gene between the *xylA* and *xylB* genes suggests that it likely regulates their transcription. This suggestion needs to be investigated.
(TIF)

S6 Fig Genomic organization of the L-arabinose utilization genes in *A*. *schaalii* DSM 15541^T^. The organism contains two kinetically distinguischable systems for L-arabinose import: the AraE L-arabinose:H+ symporter and the ATP-driven system. The two sets of transport proteins are located nearby one another, separated by the genes of the *ara* operon. The genes of the *ara* operon encode three enzymes required for arabinose catabolism: *araA* (encoding L-arabinose isomarise), *araB* (encoding L-ribulokinase) and *araD* (encoding L-ribulose-5-phosphate 4-epimerase). Upstream of the *araBDA* genes are two genes: the *araE* gene encodes a proton symporter of the MFS superfamily for the transport of arabinose into the cell and is organized as a divergent transcriptional unit with the *araR* gene encodes a LacI-type transcriptional regulator. Downstream of the *ara* operon separated by the divergently oriented *agaR* gene are the components of the ABC transporter: *G444DRAFT_00668* encodes the substrate-binding protein, *G444DRAFT_00669* encodes the ATP-binding-protein, *G444DRAFT_00670* and *G444DRAFT_00671* encode two permeases. Functional analysis is required to confirm the role of the two systems in arabinose transport.
(TIF)

S7 Fig Genomic organization of the L-rhamnose utilization genes in *A*. *schaalii* DSM 15541^T^. The *rha* operon comprises three genes *rhaDBA* encoding for enzymes mediating the canonical phosphorylated catabolic pathway for L-Rha; *rhaA* encodes L-Rha isomerase (EC 5.3.1.14 5.3.1.-); *rhaB* encodes rhamnulokinase (EC2.7.1.5); *rhaD* encodes L-rhamnulose-1-phosphate aldolase (EC 4.1.2.19). The three enzymes catalyse the conversion of L-rhamnose to dihroxyacetone phosphate (DHAP) and L-lactaldehyde. In addition, the *rhaM* gene encodes L-rhamnose mutarotase (EC 5.1.3.32), which catalyzes the interconversion of α and β anomers of L-rhamnose.
(TIF)

S8 Fig Schematic representation of the central carbohydrate metabolism in *A*. *schaalii*. All the pathways described converge with the glucose catabolic pathway at a specific point that can be fructose-6-P, pyruvate or glyceraldehyde-3-P.
(TIF)
