# Supplementary figures and images for: Draft genome sequence of Actinotignum schaalii DSM 15541T: Genetic insights into the lifestyle, cell fitness and virulence
Source: PLoS One. 2017 Dec 7;12(12):e0188914. doi: 10.1371/journal.pone.0188914 (PMC5720513; doi:10.1371/journal.pone.0188914)

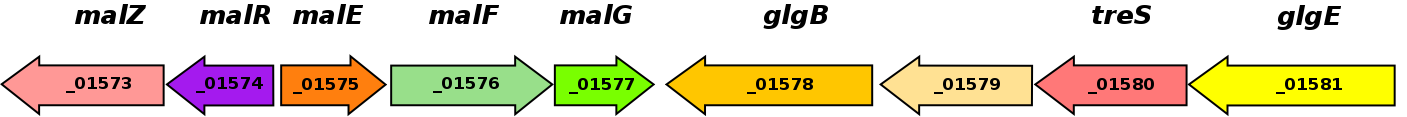

Supplement: S1 Fig — The maltose/maltodextrin ABC transporter encoded by the malEFG gene cluster. The organization of the locus within the (G444DRAFT_01575—G444DRAFT_01576—G444DRAFT_01577) gene cluster is similar to that observed in the genomes of other Actinobacteria e.g. Streptomyces coelicolor and Streptomyces erythrea. Abbreviations: malE encodes a maltose-binding protein; malF and malG encode permeases of the ABC transporter; aglA encodes α-glucosidase; malR encodes transcriptional regulator of the LacI family. Two copies of the malK genes encoding the ATPase are located elsewhere in the genome. Orthologs are shown by matching colors. (TIF) [file pone.0188914.s001.tif]

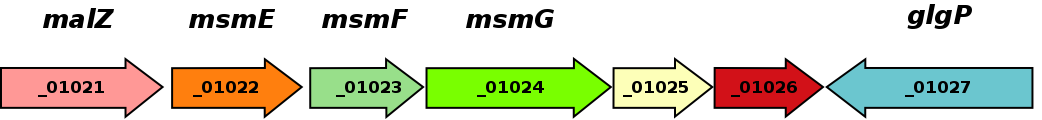

Supplement: S2 Fig — The multiple sugar ABC transporter contains msmXEFGK gene cluster. Abbreviations: malZ encodes α-amylase (EC 3.2.1.20); msmE encodes a sugar-binding protein; msmF, msmG encode two permeases; msmK encodes an ATP-binding protein located elsewhere in the genome. (TIF) [file pone.0188914.s002.tif]

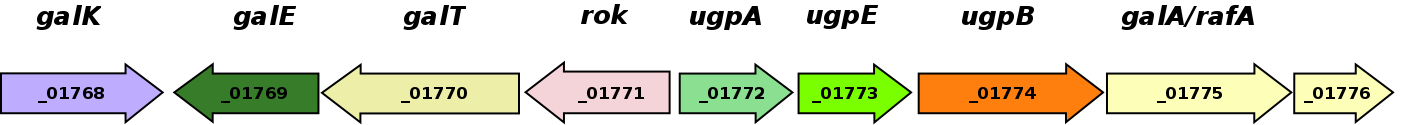

Supplement: S3 Fig — The galactose operon comprises the galRKTEM genes encoding a repressor and enzymes for the Leloir pathway for galactose metabolism. The gal opern clusters together with three genes encoding for a multiple sugar ABC transporter. Abbreviation: galR, galK, galT, galE and galM encode gal repressor (ROK family), galactokinase (EC 2.7.1.6), galactose-l-phosphate uridyltransferase (EC 2.7.7.12), UDP glucose-4-epimerase (EC 5.1.3.2) and aldose-1-epimerase (EC 5.1.3.3), respectively. The galR and galK are divergently oriented with respect to the galT and galE genes. The galM is not a part of the gal operon and was located elsewhere in the chromosome. On the opposite strand to galR and transcribed divergently are four open reading frames encoding for an ABC transporter of the CUT1 family (G444DRAFT_01772 to G444DRAFT_01774) and α-galactosidase (G444DRAFT_01775). Two importers, one in E. coli and the second in M. tuberculosis, presenting similar organization: the glycerol-3-phosphate transporter in E. coli encoded by genes (ugpA-ugpE-ugpB-ugbC) and the sugar transporter in M. tuberculosis encoded by genes (sugA-sugB-sugC-lpqY). A. schaalii transporter, however, differs from E. coli and M. tuberculosis in lacking homologs the ugpC and sugC genes, respectively. Arrows indicate direction of transcription. (TIF) [file pone.0188914.s003.tif]

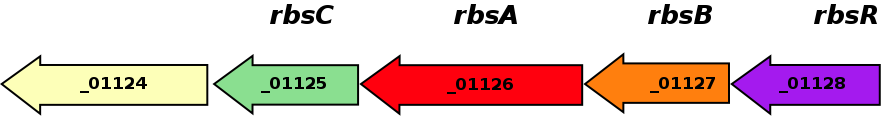

Supplement: S4 Fig — The rbsBACR gene cluster is responsible for the metabolism of ribose. The rbsBAC genes encoding for the ABC transporter belonging to the CUT2 family, where rbsB encodes a ribose-binding protein, rbsA encodes ATP-binding protein and rbsC encodes a permease. In addition to the rbsA gene, the genome harbors three rbsK genes encoding three ribokinases (EC 2.7.1.15), which specifically directs its phosphorylating activity towards d-ribose, converting this pentose sugar to ribose-5-phosphate. The transcription of the rbs gene cluster is regulated by a LacI-type regulator encoded by rbsR, located immediately upstream of rbsB. (TIF) [file pone.0188914.s004.tif]

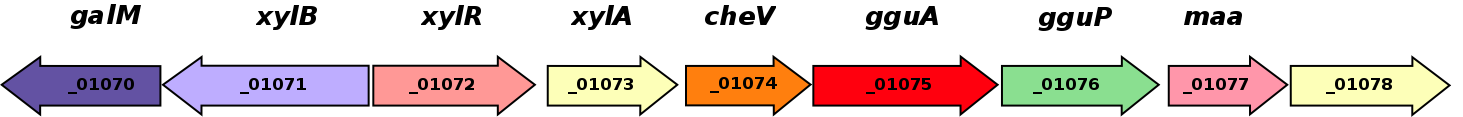

Supplement: S5 Fig — The xylose ABC transporter genes (cheV and gguAP) cluster with the xylose utilization genes xylBRA. Abbreviations: cheV encodes a xylose-binding protein; gguA encodes ATP-binding protein; gguP encodes permease of the ABC transporter; xylA encodes xylose isomerase (EC 5.3.1.5); xylB encodes xylulokinase (EC 2.7.1.17); xylR encodes transcriptional regulator of the ROK /IclR family. The products of the xylA and xylB genes together catalyze the conversion of xylose to xylulose-5-phosphate. The location of the xylR gene between the xylA and xylB genes suggests that it likely regulates their transcription. This suggestion needs to be investigated. (TIF) [file pone.0188914.s005.tif]

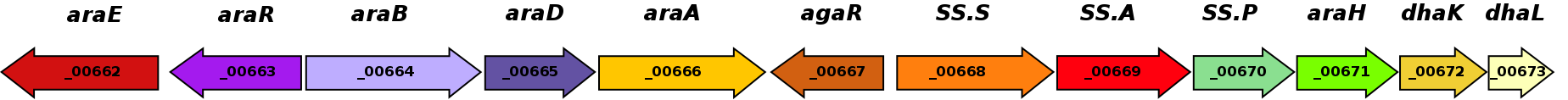

Supplement: S6 Fig — The organism contains two kinetically distinguischable systems for L-arabinose import: the AraE L-arabinose:H+ symporter and the ATP-driven system. The two sets of transport proteins are located nearby one another, separated by the genes of the ara operon. The genes of the ara operon encode three enzymes required for arabinose catabolism: araA (encoding L-arabinose isomarise), araB (encoding L-ribulokinase) and araD (encoding L-ribulose-5-phosphate 4-epimerase). Upstream of the araBDA genes are two genes: the araE gene encodes a proton symporter of the MFS superfamily for the transport of arabinose into the cell and is organized as a divergent transcriptional unit with the araR gene encodes a LacI-type transcriptional regulator. Downstream of the ara operon separated by the divergently oriented agaR gene are the components of the ABC transporter: G444DRAFT_00668 encodes the substrate-binding protein, G444DRAFT_00669 encodes the ATP-binding-protein, G444DRAFT_00670 and G444DRAFT_00671 encode two permeases. Functional analysis is required to confirm the role of the two systems in arabinose transport. (TIF) [file pone.0188914.s006.tif]

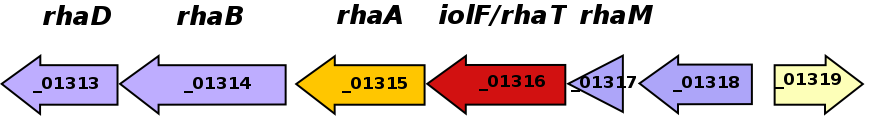

Supplement: S7 Fig — The rha operon comprises three genes rhaDBA encoding for enzymes mediating the canonical phosphorylated catabolic pathway for L-Rha; rhaA encodes L-Rha isomerase (EC 5.3.1.14 5.3.1.-); rhaB encodes rhamnulokinase (EC2.7.1.5); rhaD encodes L-rhamnulose-1-phosphate aldolase (EC 4.1.2.19). The three enzymes catalyse the conversion of L-rhamnose to dihroxyacetone phosphate (DHAP) and L-lactaldehyde. In addition, the rhaM gene encodes L-rhamnose mutarotase (EC 5.1.3.32), which catalyzes the interconversion of α and β anomers of L-rhamnose. (TIF) [file pone.0188914.s007.tif]
